# 4-(2-Methoxy­benzyl­idene)-2-phenyl-1,3-oxazol-5(4*H*)-one

**DOI:** 10.1107/S1600536809010216

**Published:** 2009-03-25

**Authors:** Abdullah Mohamed Asiri, Mehmet Akkurt, Islam Ullah Khan, Muhammad N. Arshad

**Affiliations:** a Chemistry Department, Faculty of Science, King Abdul-Aziz University, PO Box 80203, Jeddah 21589, Saudi Arabia; b Department of Physics, Faculty of Arts and Sciences, Erciyes University, 38039 Kayseri, Turkey; c Department of Chemistry, Government College University, Lahore, Pakistan

## Abstract

The title mol­ecule, C_17_H_13_NO_3_, adopts a *Z* configuration about the central olefinic bond. The 2-phenyl ring is almost coplanar with the plane of the oxazolone ring system, making a dihedral angle of 2.03 (11)°. The crystal structure is stabilized by π–π inter­actions between the oxazolone ring and phenyl ring of a neighbouring mol­ecule [centroid–centroid distance = 3.550 (3)Å], and by two weak inter­molecular C—H⋯π inter­actions. In addition, the crystal structure exhibits one weak intra­molecular C—H⋯N hydrogen bond.

## Related literature

For general background to azalactones and their biological and pharmaceutical properties, see: Cannella *et al.* (1996 ▶); Cavelier & Verducci (1995 ▶); Gelmi *et al.* (1997 ▶); Gonzalez-Martinez, Puchades, Maquieira, Ferrer, Marco & Barcelo (1999 ▶); Gottwald & Seebach (1999 ▶); Mesaik *et al.* (2004 ▶). For bond-length data, see: Allen *et al.* (1987 ▶).
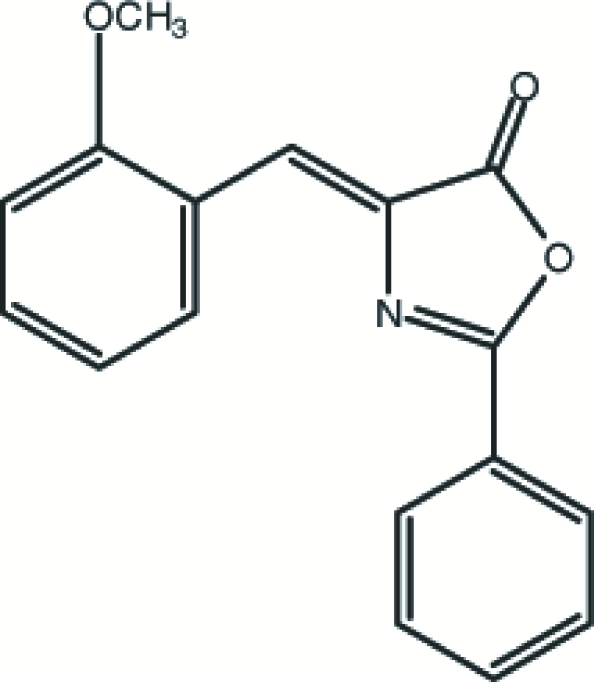


## Experimental

### 

#### Crystal data

C_17_H_13_NO_3_
*M* *_r_* = 279.28
Triclinic, 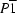

*a* = 8.8073 (6) Å
*b* = 9.6140 (6) Å
*c* = 9.8272 (6) Å
α = 66.503 (4)°
β = 67.248 (4)°
γ = 71.734 (4)°
*V* = 691.14 (8) Å^3^
*Z* = 2
Mo *K*α radiation
μ = 0.09 mm^−1^
*T* = 296 K
0.28 × 0.08 × 0.05 mm

#### Data collection

Bruker Kappa APEXII CCD diffractometer
Absorption correction: none
14582 measured reflections
3457 independent reflections
1464 reflections with *I* > 2σ(*I*)
*R*_int_ = 0.048

#### Refinement

*R*[*F* ^2^ > 2σ(*F* ^2^)] = 0.045
*wR*(*F* ^2^) = 0.133
*S* = 0.93
3457 reflections
192 parameters
H-atom parameters constrained
Δρ_max_ = 0.16 e Å^−3^
Δρ_min_ = −0.13 e Å^−3^

### 

Data collection: *APEX2* (Bruker, 2007 ▶); cell refinement: *SAINT* (Bruker, 2007 ▶); data reduction: *SAINT*; program(s) used to solve structure: *SIR97* (Altomare *et al.*, 1999 ▶); program(s) used to refine structure: *SHELXL97* (Sheldrick, 2008 ▶); molecular graphics: *ORTEP-3 for Windows* (Farrugia, 1997 ▶); software used to prepare material for publication: *WinGX* (Farrugia, 1999 ▶) and *PLATON* (Spek, 2009 ▶).

## Supplementary Material

Crystal structure: contains datablocks global, I. DOI: 10.1107/S1600536809010216/lx2096sup1.cif

Structure factors: contains datablocks I. DOI: 10.1107/S1600536809010216/lx2096Isup2.hkl

Additional supplementary materials:  crystallographic information; 3D view; checkCIF report

## Figures and Tables

**Table 1:** Hydrogen-bond geometry (Å, °)

*D*—H⋯*A*	*D*—H	H⋯*A*	*D*⋯*A*	*D*—H⋯*A*
C6—H6⋯N1	0.93	2.43	3.087 (3)	127
C17—H17*A*⋯*Cg*3^i^	0.96	2.81	3.682 (3)	151
C17—H17*C*⋯*Cg*2^ii^	0.96	2.96	3.832 (3)	151

Symmetry codes: (i) 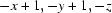; (ii) 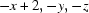. *Cg*2 is the centroid of the C1–C6 benzene ring and *Cg*3 is the centroid of the C11—C16 phenyl ring.

**Fig. 1.**
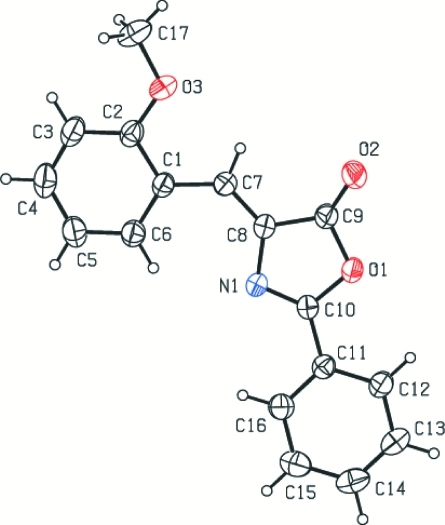
The molecular structure of the title compound with the atom numbering scheme. Displacement ellipsoids are drawn at the 30% probability level. H atoms are presented as a small spheres of arbitrary radius.

**Fig. 2.**
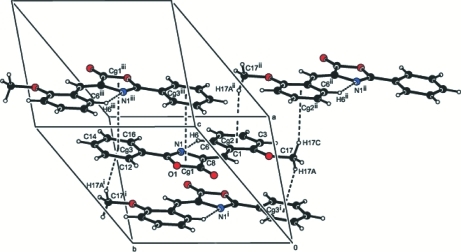
π—π, C—H···π and C—H···N interactions (dotted lines) in the title compound. Cg denotes the ring centroids. [Symmetry codes: (i) -x+1, -y+1, -z; (ii) -x+2, -y, -z; (iii) -x+1, -y+1, -z+1].

## supplementary crystallographic information

### Comment

Azalactones are a class of important heterocyclic compounds and exhibit a variety of biological and pharmaceutical properties (Mesaik *et al.*, 2004) They are also useful precursors for the synthesis of amino acids (Gottwald & Seebach, 1999), peptides (Cavelier & Verducci, 1995), heterocycles (Cannella *et al.*, 1996), biosensors (Gonzalez-Martinez *et al.*, 1999), and antitumoror antimicrobial compounds (Gelmi *et al.*, 1997). Here we report the crystal structure of the title compound, 4-(2-methoxybenzylidene)-2-phenyl-1,3-oxazol-5(4H)-one (Fig. 1).

The title molecule (Fig. 1) possesses normal geometric parameters (Allen *et al.*, 1987) and adopts a *Z* configuration about the central olefinic bond. The C11–C16 phenyl ring makes a dihedral angle of 2.03 (11) ° with the plane of the oxazolone ring system. The molecular packing (Fig. 2) is stabilized by intermolecular π—π interactions between the oxazolone ring and phenyl ring of neighbouring molecules, with a Cg1···Cg3^iii^ distance of 3.550 (3) Å (Cg1 and Cg3 are the centroids of the O1/C10/N1/C8/C9 oxazolone ring and the C11—C16 phenyl ring; symmetry code as in Fig, 2). The crystal packing is further stabilized by two intermolecular C—H···π interactions; one between the H atom of methoxy group and the phenyl ring of a neighbouring molecule, a second between the H atom of methoxy group and the methoxyphenyl ring of an adjacent molecule, respectively (Fig. 2 and Table 1; Cg2 is the centroid of the C1–C6 benzene ring, symmetry code as in Fig, 2). Additionally, there is one intramolecular C—H···N hydrogen bond between a benzene—H atom and the N atom of oxazolone ring (Table 1 and Fig. 2).

### Experimental

Anhydrous sodium acetate (2.1 g, 25.3 mmol) was added to a solution of 2-methoxybenzaldehyde (3.5 g, 25.7 mmol) and hippuric acid (7.7 g, 31.1 mmol) in acetic anhydride (2.1 g, 20.6 mmol). The reaction mixture was heated to 353 K and stirred under reflux conditions for the appropriate time 2 h. The reaction mixture was cooled to room temperature and ethanol (10 ml) was added. The mixture was stirred for 10 min until a yellow solid precipitated. The mixture was allowed to stand overnight, and then it was cooled in an ice bath. The crude azalactones were obtained after filtration and washing with hot water. Recrystallization from acetone/water afforded the pure azalactones as yellow crystals. [Yield (5.79 g, 91%), m.p. 440–441 K]. IR (cm^-1^) 1769 (C═O),1648 (C═C).

### Refinement

All H atoms were positioned geometrically with C—H = 0.93 and 0.96 Å and refined using a riding approximation model with *U*_iso_(H) = 1.2 or 1.5*U*_eq_(C).

### Crystal data

**Table:**

C_17_H_13_NO_3_	*Z* = 2
*M**_r_* = 279.28	*F*(000) = 292
Triclinic, *P*1	*D*_x_ = 1.342 Mg m^−^^3^
Hall symbol: -P 1	Mo *K*α radiation, λ = 0.71073 Å
*a* = 8.8073 (6) Å	Cell parameters from 1969 reflections
*b* = 9.6140 (6) Å	θ = 2.4–22.4°
*c* = 9.8272 (6) Å	µ = 0.09 mm^−^^1^
α = 66.503 (4)°	*T* = 296 K
β = 67.248 (4)°	Prism, yellow
γ = 71.734 (4)°	0.28 × 0.08 × 0.05 mm
*V* = 691.14 (8) Å^3^	

### Data collection

**Table:**

Bruker Kappa APEXII CCD diffractometer	1464 reflections with *I* > 2σ(*I*)
Radiation source: sealed tube	*R*_int_ = 0.048
graphite	θ_max_ = 28.5°, θ_min_ = 2.4°
Detector resolution: 10.0 pixels mm^-1^	*h* = −11→11
φ and ω scans	*k* = −12→12
14582 measured reflections	*l* = −13→13
3457 independent reflections	

### Refinement

**Table:**

Refinement on *F*^2^	Secondary atom site location: difference Fourier map
Least-squares matrix: full	Hydrogen site location: inferred from neighbouring sites
*R*[*F*^2^ > 2σ(*F*^2^)] = 0.045	H-atom parameters constrained
*wR*(*F*^2^) = 0.133	*w* = 1/[σ^2^(*F*_o_^2^) + (0.0583*P*)^2^] where *P* = (*F*_o_^2^ + 2*F*_c_^2^)/3
*S* = 0.93	(Δ/σ)_max_ = 0.001
3457 reflections	Δρ_max_ = 0.16 e Å^−^^3^
192 parameters	Δρ_min_ = −0.13 e Å^−^^3^
0 restraints	Extinction correction: *SHELXL97* (Sheldrick, 2008), FC^*^=KFC[1+0.001XFC^2^Λ^3^/SIN(2Θ)]^-1/4^
Primary atom site location: structure-invariant direct methods	Extinction coefficient: 0.008 (3)

### Special details

**Table:**

Geometry. Bond distances, angles *etc*. have been calculated using the rounded fractional coordinates. All su's are estimated from the variances of the (full) variance-covariance matrix. The cell e.s.d.'s are taken into account in the estimation of distances, angles and torsion angles
Refinement. Refinement on *F*^2^ for ALL reflections except those flagged by the user for potential systematic errors. Weighted *R*-factors *wR* and all goodnesses of fit *S* are based on *F*^2^, conventional *R*-factors *R* are based on *F*, with *F* set to zero for negative *F*^2^. The observed criterion of *F*^2^ > σ(*F*^2^) is used only for calculating -*R*-factor-obs *etc*. and is not relevant to the choice of reflections for refinement. *R*-factors based on *F*^2^ are statistically about twice as large as those based on *F*, and *R*-factors based on ALL data will be even larger.

### Fractional atomic coordinates and isotropic or equivalent isotropic displacement parameters (Å^2^)

**Table:**

	*x*	*y*	*z*	*U*_iso_*/*U*_eq_	
O1	0.25166 (15)	0.44719 (14)	0.39300 (14)	0.0569 (5)	
O2	0.21889 (18)	0.24891 (17)	0.34504 (18)	0.0822 (6)	
O3	0.71016 (18)	0.05676 (17)	−0.02406 (16)	0.0758 (6)	
N1	0.51111 (18)	0.48138 (17)	0.23057 (16)	0.0496 (6)	
C1	0.7332 (2)	0.2733 (2)	0.0149 (2)	0.0506 (7)	
C2	0.8036 (2)	0.1640 (2)	−0.0653 (2)	0.0585 (8)	
C3	0.9578 (3)	0.1700 (3)	−0.1783 (2)	0.0728 (9)	
C4	1.0418 (3)	0.2832 (3)	−0.2108 (3)	0.0812 (9)	
C5	0.9784 (3)	0.3890 (3)	−0.1317 (2)	0.0741 (9)	
C6	0.8245 (3)	0.3836 (2)	−0.0200 (2)	0.0613 (8)	
C7	0.5675 (2)	0.2690 (2)	0.1242 (2)	0.0534 (7)	
C8	0.4736 (2)	0.3592 (2)	0.2139 (2)	0.0494 (7)	
C9	0.3054 (3)	0.3362 (2)	0.3179 (2)	0.0570 (8)	
C10	0.3815 (2)	0.5263 (2)	0.3330 (2)	0.0469 (6)	
C11	0.3562 (2)	0.6487 (2)	0.3944 (2)	0.0496 (7)	
C12	0.2070 (3)	0.6867 (2)	0.5026 (2)	0.0623 (8)	
C13	0.1867 (3)	0.8024 (3)	0.5607 (3)	0.0762 (9)	
C14	0.3129 (4)	0.8808 (3)	0.5107 (3)	0.0779 (10)	
C15	0.4617 (3)	0.8459 (3)	0.4015 (3)	0.0760 (10)	
C16	0.4826 (3)	0.7300 (2)	0.3435 (2)	0.0627 (8)	
C17	0.7702 (3)	−0.0552 (3)	−0.1038 (3)	0.0876 (10)	
H3	1.00400	0.09810	−0.23150	0.0870*	
H4	1.14440	0.28850	−0.28830	0.0970*	
H5	1.03870	0.46320	−0.15340	0.0890*	
H6	0.78080	0.45550	0.03320	0.0740*	
H7	0.51770	0.19180	0.13430	0.0640*	
H12	0.12020	0.63380	0.53610	0.0750*	
H13	0.08670	0.82700	0.63400	0.0910*	
H14	0.29880	0.95860	0.55060	0.0940*	
H15	0.54730	0.90020	0.36740	0.0910*	
H16	0.58250	0.70640	0.26970	0.0750*	
H17A	0.78080	−0.00370	−0.21230	0.1310*	
H17B	0.69250	−0.12400	−0.06060	0.1310*	
H17C	0.87760	−0.11310	−0.09190	0.1310*	

### Atomic displacement parameters (Å^2^)

**Table:**

	*U*^11^	*U*^22^	*U*^33^	*U*^12^	*U*^13^	*U*^23^
O1	0.0465 (8)	0.0591 (8)	0.0597 (8)	−0.0112 (7)	−0.0015 (6)	−0.0266 (7)
O2	0.0612 (10)	0.0754 (11)	0.1086 (12)	−0.0262 (9)	0.0020 (8)	−0.0443 (9)
O3	0.0732 (10)	0.0836 (11)	0.0839 (10)	−0.0087 (9)	−0.0127 (8)	−0.0548 (9)
N1	0.0455 (10)	0.0547 (10)	0.0470 (9)	−0.0084 (8)	−0.0074 (8)	−0.0216 (8)
C1	0.0443 (12)	0.0614 (13)	0.0448 (10)	−0.0030 (10)	−0.0130 (9)	−0.0211 (10)
C2	0.0491 (13)	0.0736 (15)	0.0529 (12)	0.0025 (11)	−0.0192 (10)	−0.0271 (11)
C3	0.0546 (14)	0.1005 (19)	0.0614 (13)	0.0044 (14)	−0.0130 (11)	−0.0424 (13)
C4	0.0519 (14)	0.116 (2)	0.0599 (14)	−0.0092 (15)	−0.0056 (11)	−0.0282 (15)
C5	0.0538 (14)	0.0970 (18)	0.0667 (14)	−0.0201 (13)	−0.0109 (12)	−0.0236 (13)
C6	0.0533 (13)	0.0743 (15)	0.0558 (12)	−0.0103 (12)	−0.0134 (10)	−0.0243 (11)
C7	0.0508 (12)	0.0569 (13)	0.0542 (11)	−0.0078 (10)	−0.0138 (10)	−0.0230 (10)
C8	0.0442 (12)	0.0517 (12)	0.0486 (11)	−0.0064 (10)	−0.0102 (9)	−0.0179 (10)
C9	0.0504 (13)	0.0538 (13)	0.0639 (13)	−0.0091 (11)	−0.0088 (10)	−0.0244 (11)
C10	0.0417 (11)	0.0501 (12)	0.0449 (10)	−0.0090 (10)	−0.0112 (9)	−0.0126 (9)
C11	0.0476 (12)	0.0492 (12)	0.0504 (11)	−0.0018 (10)	−0.0184 (9)	−0.0162 (10)
C12	0.0587 (14)	0.0646 (14)	0.0605 (12)	−0.0086 (11)	−0.0091 (10)	−0.0275 (11)
C13	0.0818 (18)	0.0753 (16)	0.0744 (15)	−0.0038 (14)	−0.0150 (13)	−0.0430 (13)
C14	0.100 (2)	0.0622 (15)	0.0858 (17)	0.0001 (15)	−0.0409 (15)	−0.0367 (13)
C15	0.0810 (18)	0.0670 (15)	0.0942 (18)	−0.0148 (13)	−0.0392 (15)	−0.0266 (14)
C16	0.0569 (14)	0.0641 (14)	0.0705 (13)	−0.0072 (11)	−0.0207 (11)	−0.0264 (12)
C17	0.107 (2)	0.0841 (17)	0.0864 (16)	0.0101 (15)	−0.0377 (15)	−0.0545 (15)

### Geometric parameters (Å, °)

**Table:**

O1—C9	1.396 (2)	C11—C16	1.380 (3)
O1—C10	1.378 (2)	C12—C13	1.378 (3)
O2—C9	1.192 (3)	C13—C14	1.361 (5)
O3—C2	1.358 (3)	C14—C15	1.380 (4)
O3—C17	1.431 (3)	C15—C16	1.377 (3)
N1—C8	1.398 (3)	C3—H3	0.9300
N1—C10	1.285 (2)	C4—H4	0.9300
C1—C2	1.409 (3)	C5—H5	0.9300
C1—C6	1.386 (3)	C6—H6	0.9300
C1—C7	1.441 (3)	C7—H7	0.9300
C2—C3	1.383 (3)	C12—H12	0.9300
C3—C4	1.371 (4)	C13—H13	0.9300
C4—C5	1.375 (4)	C14—H14	0.9300
C5—C6	1.376 (3)	C15—H15	0.9300
C7—C8	1.345 (3)	C16—H16	0.9300
C8—C9	1.464 (3)	C17—H17A	0.9600
C10—C11	1.448 (3)	C17—H17B	0.9600
C11—C12	1.385 (3)	C17—H17C	0.9600
			
O1···N1	2.260 (2)	C4···H17C^vii^	3.0100
O2···C14^i^	3.236 (3)	C5···H17C^vii^	2.9800
O2···C12^ii^	3.411 (3)	C8···H6	2.8100
O1···H4^iii^	2.8000	C14···H17A^iv^	3.0300
O1···H12	2.4500	C15···H17A^iv^	3.0200
O2···H7	2.7000	C16···H4^viii^	3.0700
O2···H14^i^	2.7800	C17···H3	2.5300
O2···H12^ii^	2.7900	H3···C17	2.5300
O2···H13^ii^	2.9200	H3···H17A	2.3700
O3···H7	2.2700	H3···H17C	2.2900
N1···O1	2.260 (2)	H4···O1^ix^	2.8000
N1···C6	3.087 (3)	H4···C16^viii^	3.0700
N1···H6	2.4300	H4···H16^viii^	2.5000
N1···H16	2.6300	H6···N1	2.4300
C1···C10^iv^	3.553 (3)	H6···C8	2.8100
C2···C10^iv^	3.532 (3)	H7···O2	2.7000
C5···C9^iv^	3.481 (4)	H7···O3	2.2700
C6···N1	3.087 (3)	H7···H17B^x^	2.5600
C6···C9^iv^	3.392 (3)	H12···O1	2.4500
C6···C8^iv^	3.561 (3)	H12···O2^ii^	2.7900
C8···C14^v^	3.524 (4)	H13···O2^ii^	2.9200
C8···C6^iv^	3.561 (3)	H14···O2^vi^	2.7800
C9···C5^iv^	3.481 (4)	H16···N1	2.6300
C9···C6^iv^	3.392 (3)	H16···H4^viii^	2.5000
C10···C16^v^	3.530 (3)	H17A···C3	2.7900
C10···C2^iv^	3.532 (3)	H17A···H3	2.3700
C10···C1^iv^	3.553 (3)	H17A···C14^iv^	3.0300
C12···O2^ii^	3.411 (3)	H17A···C15^iv^	3.0200
C14···C8^v^	3.524 (4)	H17B···H7^x^	2.5600
C14···O2^vi^	3.236 (3)	H17C···C3	2.7400
C16···C10^v^	3.530 (3)	H17C···H3	2.2900
C3···H17A	2.7900	H17C···C4^vii^	3.0100
C3···H17C	2.7400	H17C···C5^vii^	2.9800
			
C9—O1—C10	105.44 (16)	C14—C15—C16	119.5 (3)
C2—O3—C17	119.06 (18)	C11—C16—C15	120.4 (2)
C8—N1—C10	105.35 (16)	C2—C3—H3	120.00
C2—C1—C6	118.16 (17)	C4—C3—H3	120.00
C2—C1—C7	118.56 (17)	C3—C4—H4	119.00
C6—C1—C7	123.23 (18)	C5—C4—H4	119.00
O3—C2—C1	115.60 (17)	C4—C5—H5	120.00
O3—C2—C3	123.94 (19)	C6—C5—H5	120.00
C1—C2—C3	120.5 (2)	C1—C6—H6	119.00
C2—C3—C4	119.2 (2)	C5—C6—H6	119.00
C3—C4—C5	121.7 (3)	C1—C7—H7	115.00
C4—C5—C6	119.1 (3)	C8—C7—H7	115.00
C1—C6—C5	121.38 (19)	C11—C12—H12	120.00
C1—C7—C8	130.09 (18)	C13—C12—H12	120.00
N1—C8—C7	129.85 (18)	C12—C13—H13	120.00
N1—C8—C9	108.50 (16)	C14—C13—H13	120.00
C7—C8—C9	121.64 (18)	C13—C14—H14	120.00
O1—C9—O2	121.5 (2)	C15—C14—H14	120.00
O1—C9—C8	104.65 (18)	C14—C15—H15	120.00
O2—C9—C8	133.90 (18)	C16—C15—H15	120.00
O1—C10—N1	116.07 (17)	C11—C16—H16	120.00
O1—C10—C11	116.07 (16)	C15—C16—H16	120.00
N1—C10—C11	127.85 (18)	O3—C17—H17A	110.00
C10—C11—C12	121.32 (19)	O3—C17—H17B	109.00
C10—C11—C16	119.45 (17)	O3—C17—H17C	109.00
C12—C11—C16	119.23 (18)	H17A—C17—H17B	109.00
C11—C12—C13	120.2 (2)	H17A—C17—H17C	109.00
C12—C13—C14	120.0 (3)	H17B—C17—H17C	109.00
C13—C14—C15	120.6 (3)		
			
C10—O1—C9—O2	−179.7 (2)	C3—C4—C5—C6	−1.8 (4)
C10—O1—C9—C8	0.43 (19)	C4—C5—C6—C1	0.5 (4)
C9—O1—C10—N1	−0.3 (2)	C1—C7—C8—N1	−2.0 (3)
C9—O1—C10—C11	−179.54 (16)	C1—C7—C8—C9	179.55 (19)
C17—O3—C2—C1	177.88 (19)	N1—C8—C9—O1	−0.5 (2)
C17—O3—C2—C3	−1.9 (3)	N1—C8—C9—O2	179.7 (2)
C10—N1—C8—C7	−178.4 (2)	C7—C8—C9—O1	178.32 (17)
C10—N1—C8—C9	0.3 (2)	C7—C8—C9—O2	−1.5 (4)
C8—N1—C10—O1	0.0 (2)	O1—C10—C11—C12	−2.4 (3)
C8—N1—C10—C11	179.13 (18)	O1—C10—C11—C16	178.04 (17)
C6—C1—C2—O3	178.68 (18)	N1—C10—C11—C12	178.42 (19)
C6—C1—C2—C3	−1.6 (3)	N1—C10—C11—C16	−1.1 (3)
C7—C1—C2—O3	−3.8 (3)	C10—C11—C12—C13	179.2 (2)
C7—C1—C2—C3	175.91 (19)	C16—C11—C12—C13	−1.2 (3)
C2—C1—C6—C5	1.2 (3)	C10—C11—C16—C15	−179.4 (2)
C7—C1—C6—C5	−176.2 (2)	C12—C11—C16—C15	1.1 (3)
C2—C1—C7—C8	−179.85 (19)	C11—C12—C13—C14	0.6 (4)
C6—C1—C7—C8	−2.5 (3)	C12—C13—C14—C15	0.2 (4)
O3—C2—C3—C4	−180.0 (2)	C13—C14—C15—C16	−0.4 (4)
C1—C2—C3—C4	0.3 (3)	C14—C15—C16—C11	−0.3 (4)
C2—C3—C4—C5	1.4 (4)		

Symmetry codes: (i) *x*, *y*−1, *z*; (ii) −*x*, −*y*+1, −*z*+1; (iii) *x*−1, *y*, *z*+1; (iv) −*x*+1, −*y*+1, −*z*; (v) −*x*+1, −*y*+1, −*z*+1; (vi) *x*, *y*+1, *z*; (vii) −*x*+2, −*y*, −*z*; (viii) −*x*+2, −*y*+1, −*z*; (ix) *x*+1, *y*, *z*−1; (x) −*x*+1, −*y*, −*z*.

### Hydrogen-bond geometry (Å, °)

**Table:**

*D*—H···*A*	*D*—H	H···*A*	*D*···*A*	*D*—H···*A*
C6—H6···N1	0.93	2.43	3.087 (3)	127
C17—H17A···Cg3^iv^	0.96	2.81	3.682 (3)	151
C17—H17C···Cg2^vii^	0.96	2.96	3.832 (3)	151

Symmetry codes: (iv) −*x*+1, −*y*+1, −*z*; (vii) −*x*+2, −*y*, −*z*.
